# Supplementary material for: Towards omics-based predictions of planktonic functional composition from environmental data
Source: Nat Commun. 2021 Jul 16;12:4361. doi: 10.1038/s41467-021-24547-1 (PMC8285379; doi:10.1038/s41467-021-24547-1)
Supplement: Supplementary file 1 — Supplementary Information [file 41467_2021_24547_MOESM1_ESM.pdf]

SUPPLEMENTARY INFORMATION

Supplementary notes

I- Detection of the *rare biosphere*

By defining dark protein functional clusters (PFCs) as clusters of protein sequences which are all functionally unannotated and taxonomically unannotated under the phylum level, we selected abundant PFCs, probably missing most of the "*rare biosphere*" [1] (See main text). To test the impact of our definition, we investigated the abundance of dark PFCs using other taxonomic rank thresholds to define microbial dark PFCs.
12,895 PFCs were only composed of proteins with no functional annotation in KEGG and eggNOG databases, and had no taxonomic annotation under the class level. The abundance of these 12,895 PFCs was significantly different from the one of the rest of PFCs in 88 samples over 93 (Wilcoxon test, p-value <0.05). Their median abundance was lower in 32 of these 88 samples, and higher in the 56 others.
20,166 PFCs were only composed of proteins with no functional annotation in KEGG and eggNOG databases, and had no taxonomic annotation under the order level. The abundance of these 20,166 PFCs was significantly different from the one of the rest of PFCs in 84 samples over 93 (Wilcoxon test, p-value <0.05). Their median abundance was lower in 65 of these 84 samples, and higher in the 19 others.
32,737 PFCs were only composed of proteins with no functional annotation in KEGG and eggNOG databases, and had no taxonomic annotation under the family level. The abundance of these 32,737 PFCs was significantly different from the one of the rest of PFCs in all samples (Wilcoxon test, p-value <0.05), and their median abundance was lower in all samples except one, a surface sample from the Indian Ocean (TARA_064).
In this way, step by step considering PFCs taxonomically unannotated under the class, order and family level as microbial dark omics led to a decrease of median abundance of dark PFCs. It then seems that the "*rare biosphere*" was better detected when including unidentified lineages of known class, order or family, than when using only unidentified lineages of known phylum. As stated in the main text, this can be explained by the lack of knowledge about the abundant *Archaea* and *Candidatus Marinimicrobia* phyla. Although, we could also argue that using MAGs might not be the best way to study the "*rare biosphere*". Indeed, the binning of contigs into MAGs relying mainly on co-abundance profiles [2–4], it is likely that organisms with very low and erratic abundances over samples could be missed in the binning steps.

II- Global biogeography of protein functional clusters associated with models showing R^2 values over 0.25

In the main text we focused on the biogeography of 14,585 highly linked to the environment PFCs (hlePFCs), to facilitate the detection of PFCs, metabolic pathways and taxonomic groups that were the most related to particular environmental conditions. Here, we present a similar biogeographical investigation of PFC abundance, but this time focusing on the 130,650 PFCs that were associated with models showing R^2 values over 0.25, which corresponds to 55.9% of the 233,756 PFCs of our study, and 444,785 (59.1%) of the 757457

proteins of our sequence similarity network. Methods used for this biogeographical study were the same as the ones described in paragraph *Biogeography of protein functional clusters (PFCs) linked to environmental gradients*.

II. a- Canonical correspondence analysis on PFCs associated with models showing R^2 values over 0.25

22 environmental variables were selected through backward and forward AIC-based stepwise selection in our final CCA model on the 130,650 PFCs associated with models showing R^2 values over 0.25: biogeographical provinces, season moment, maximum depth of sampling, fluorescence, calcite saturation state, annual oxygen saturation, oxygen saturation seasonal anomaly, dissolved oxygen seasonal anomaly, latitude, depth of the euphotic zone, sunshine duration, longitude, salinity, lyapunov exponent, bathymetry, annual density, sea surface temperature gradient, depth of the maximum Brunt-Väisälä frequency, ammonium at 5m depth, moon phase proportion, ocean region and size fraction. The final CCA model was significant (p-value<0.001), and had an adjusted R^2 value of 57.7%.

The axis (12.75% of explained variance) opposed two samples with high fluorescence and oxygen saturation anomaly, both coming from station 93 (CCA1<0), from the rest of samples (Figure S5A). The second axis (7.29% of variance explained) opposed deep samples (CCA2>0) from shallow samples (CCA2<0) (Figure S5A). The third axis of the CCA opposed dense and saline samples from the Mediterranean Sea (CCA3<0) to samples from the Indian Ocean (CCA3>0) (Figure S5B). The fourth axis (5.82% of variance explained) opposed polar samples (CCA4>0) to the rest of samples (Figure S5B). This way, the CCA triplot corresponding to axes 3 and 4 (Figure S5B) was very similar to the one presented in Figure 2, with axes opposing the same samples, while the first two dimensions of this CCA allowed for the differentiation of 2 samples as strong outliers, while they were not identified as such in Figure 2. These two samples came from the surface and deep chlorophyll maximum of Station 93, which is the closest one from the Chilean coastline in our dataset, and located in an upwelling area.
Considering the important similarity between axes 3 and 4 of the CCA presented here and axes 1 and 2 of the CCA on hlePFCs presented in the main text, we will focus here on investigating the outlying position of the two samples from station 93.

II. b- Investigating the outlying position of station 93 in details

Combining CCA results with functional annotations of PFCs, we observed no metabolic pathway particularly over-represented among PFCs associated with the two samples from station 93 (Figure S6). Staurosporine biosynthesis was the metabolic pathway that was the most associated with negative values on CCA1 (Figure S6), but was detected only 12 times among the more than 400,000 proteins present in the CCA space.

Combining CCA results with taxonomic annotations of PFCs, we were able to identify a strong over-representation of PFCs annotated to the genus *Pseudoalteromonas* on the negative side of CCA1 (Figure S7). 2,836 PFCs had coordinates below -3 on CCA1, implying that they had a strong association with the two outlier samples. These 2,836 PFCs

contained 8,364 proteins, belonging to 71 distinct MAGs. 6,293 (75.2%) of these 8,364 proteins came from MAGs annotated to the *Pseudoalteromonas* genus, while the second most represented genus in this set of proteins was Vibrio, with only 108 proteins (1.3%). 5 MAGs of *Pseudoalteromonas* contributed each to more than 1000 proteins among PFCs located under -3 on CCA1: TARA_ASW_MAG_00044, TARA_MED_MAG_00139, TARA_MED_MAG_00142, TARA_PSE_MAG_00132 and TARA_PSE_MAG_00144.

II. c- Why was this pattern absent from the CCA on the hlePFCs ?

Among models associated with these 2836 PFCs, the mean R^2 was of 0.32, and only 21 (0.7%) of them were hlePFCs (*i.e.* had R^2 values above 0.5). Hence, less than 1% of the PFCs driving the outlying position of samples from station 93 were included in the hlePFCs CCA, which explains why the pattern was not observable on Figure 2. The relatively low values of R^2 values associated with these 2,836 PFCs can be explained by the fact that they were mostly abundant in only 2 samples over 93, and hence the presence of one or both those samples among out-of-bag samples during cross-validation or even in test sets could drastically influence the quality of prediction of their abundance. By including more samples corresponding to similar environmental conditions (*i.e.* coastal and/or upwelling areas), it is likely that a similar pattern, if it was conserved, would have led to higher R^2 values among concerned PFCs. We would then be able to confidently associate the *Pseudoalteromonas* related PFCs with coastal upwelling conditions. However, samples from a station with similar conditions could also show a very distinct PFC composition compared to station 93, and in this case the R^2 values of the models associated with PFCs related to this station would decrease. The pattern that we observe here would then be spatially restricted to station 93 (and eventually temporally restricted to the moment of *Tara Oceans* sampling), and the PFCs identified here would not necessarily be linked with the upwelling or coastal conditions, but more to the geography. The fact that this pattern is only related to one station prevents us from building any strong conclusions on the potential ecological role of these PFCs. It highlights how the potential of our approach to pinpoint PFCs associated with particular environmental conditions is highly dependent on the amount of samples included, and the diversity of conditions that they represent.

References

1. Lynch MDJ, Neufeld JD. Ecology and exploration of the rare biosphere. *Nat Rev Microbiol* 2015; **13**: 217–229.

2. Delmont TO, Quince C, Shaiber A, Esen OC, Lee ST, Rappé MS, et al. Nitrogen-fixing populations of Planctomycetes and Proteobacteria are abundant in surface ocean metagenomes. *Nat Microbiol* 2018; **3**: 804–813.

3. Parks DH, Rinke C, Chuvochina M, Chaumeil P-A, Woodcroft BJ, Evans PN, et al. Recovery of nearly 8,000 metagenome-assembled genomes substantially expands the

tree of life. *Nat Microbiol* 2017; **2**: 1533–1542.

4. Tully BJ, Graham ED, Heidelberg JF. The reconstruction of 2,631 draft metagenome-assembled genomes from the global oceans. *Sci Data* 2018; **5**: 170203.

Supplementary figures

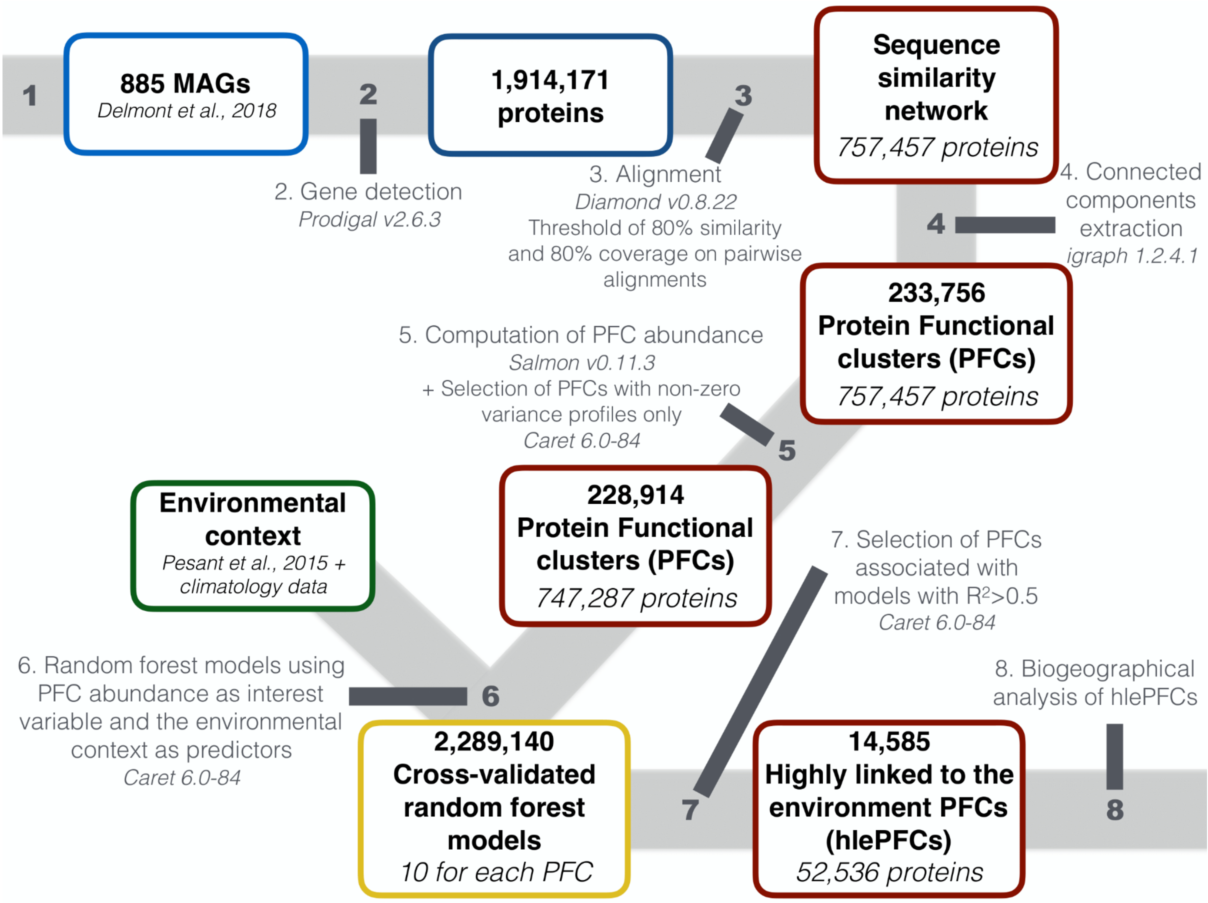

Supplementary Figure 1*: Flowchart of the bioinformatic pipeline developed for this study. All bash and R codes used for these different steps are available at https://github.com/EmileFaure/MAGsProteinFunctionalClusters. The only bioinformatic step missing from this flowchart is the functional annotation, which was achieved through eggNOG mapper v4.5.1 and Kofamscan (please refer to Methods for details and references). Any set of genomes, metagenomes, transcriptomes or metatranscriptomes could be used at step 1. At step 6, 10 models are computed on 10 distinct random training sets including 75% of the samples each. At step 7, the selection is made on the mean R^2 over the 10 computed models for each protein functional clusters (PFC).*

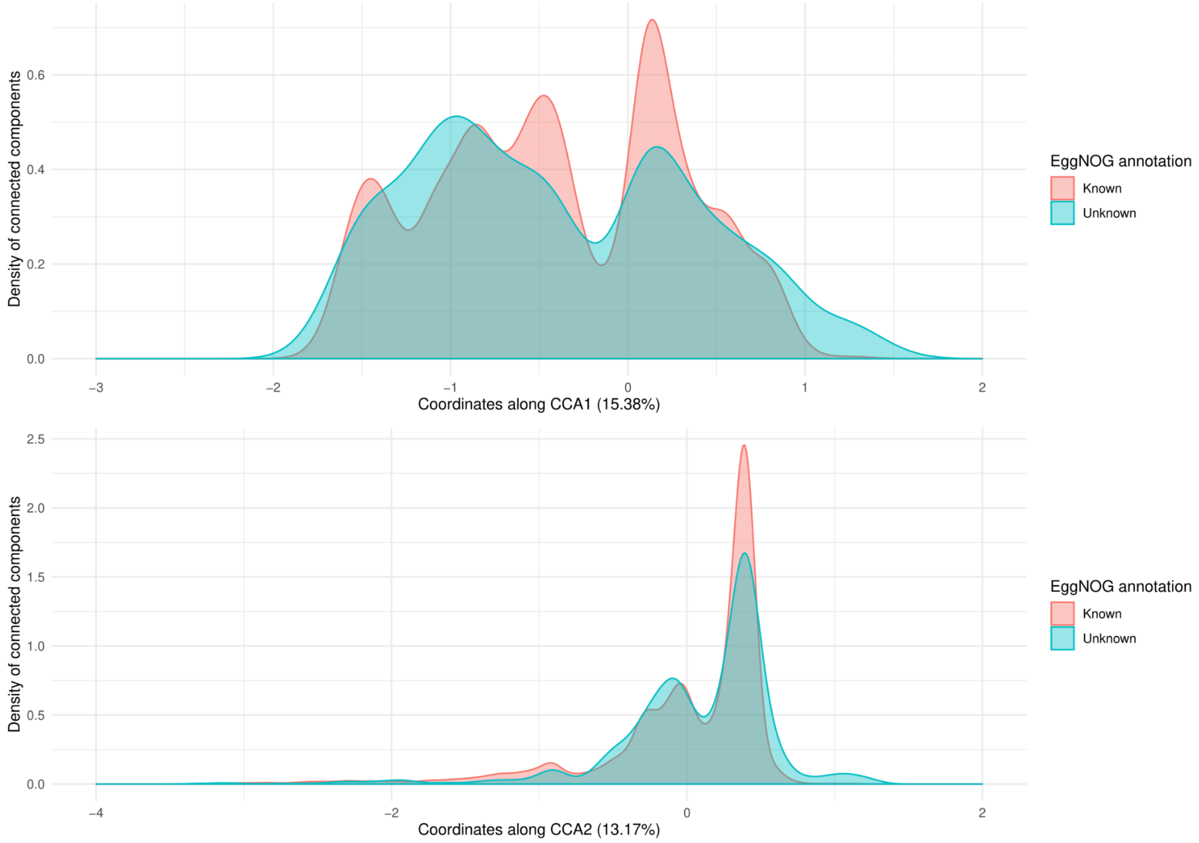

Supplementary Figure 2*: Density plots illustrating the proportions of functional known and unknown hlePFCs along CCA1 and CCA2 axes, based on eggNOG annotations (i.e. a known hlePFC corresponds to a protein cluster in which at least one sequence has an eggNOG assignment; an unknown hlePFC corresponds to a protein cluster without any eggNOG assignment). The mean hlePFC density was of 0.25 along CCA1 (standard deviation = 0.2, maximum = 0.71), and 0.2 along CCA2 (standard deviation = 0.42, maximum = 2.46). The mean difference in density between functional known and unknown hlePFCs along CCA1 was of 0.02 (standard deviation = 0.09, maximum = 0.28). The mean density difference between known and unknown hlePFCs along CCA2 was 0.005 (standard deviation = 0.34, maximum = 1.9).*

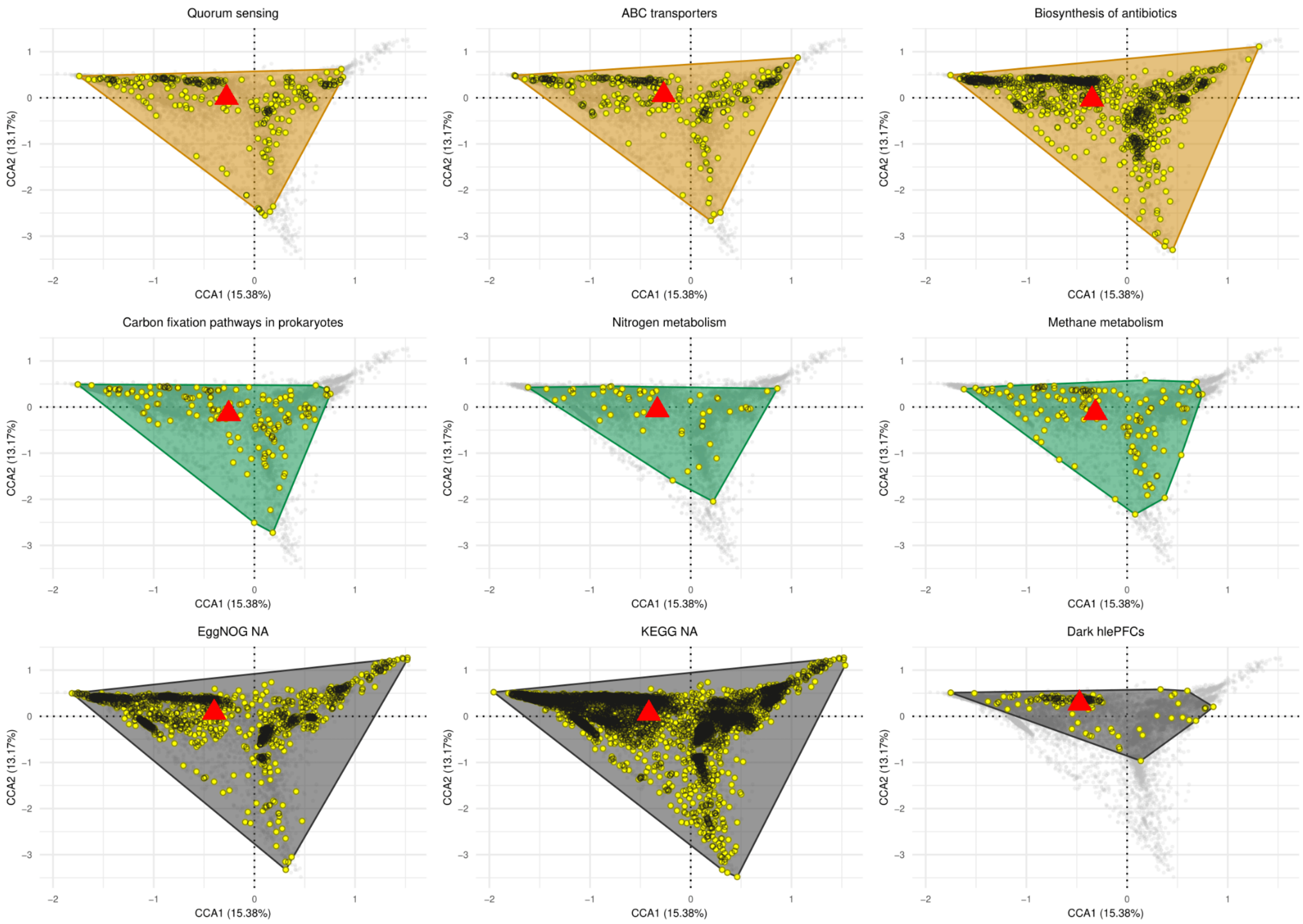

Supplementary Figure 3*: Convex hulls englobing all hlePFCs associated to different pathways in the CCA two-dimensional space. On each graph, grey dots represent hlePFCs that are not associated to the focal pathway, while yellow dots represent hlePFCs containing at least one sequence associated to the focal pathway. Convex hulls were drawn in different colors depending on the type of pathway. We selected three pathways linked to inter-organisms interactions, in orange hulls, and three pathways related to biogeochemical functions in green hulls. Finally, three black convex hulls englobing all functional unknown PFCs were represented, one for KEGG annotations, one for eggNOG ones, and one corresponding to dark hlePFCs (no functional annotation in both databases and no taxonomic annotation under the Phylum level).*

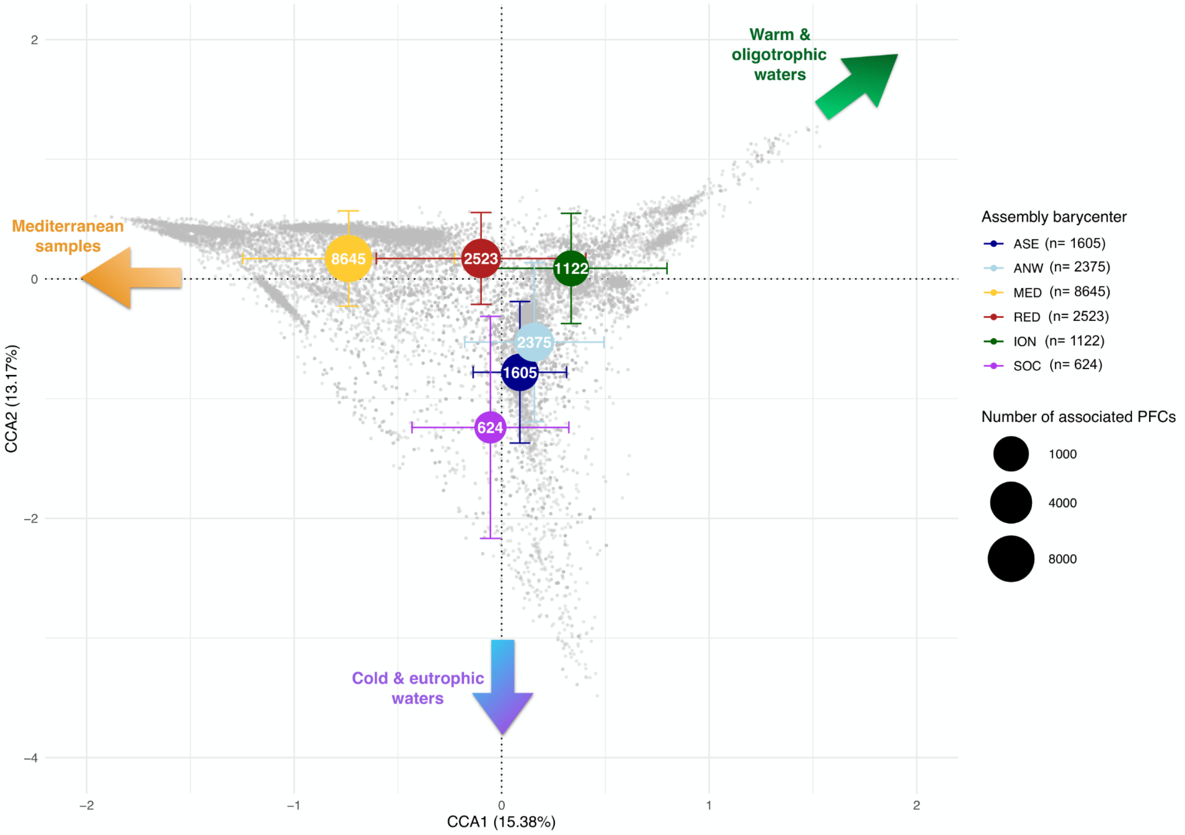

Supplementary Figure 4*: Barycenters of the protein functional clusters highly linked to environmental gradients (hlePFCs) from 6 selected assemblies in the canonical correspondence analysis (CCA) space. These assemblies were selected because they had the most peripheral barycenters' positions in the CCA space. Error bars correspond to the standard deviations of hlePFC positions around their barycenters on CCA1 and CCA2 axis for each assembly. Size of barycenters represent the number of associated hlePFCs for each assembly, with the exact corresponding values written in white in each barycenter as well as in the legend. Colored arrows indicate the environmental conditions associated with the different zones of the CCA space (See Figure 2).*

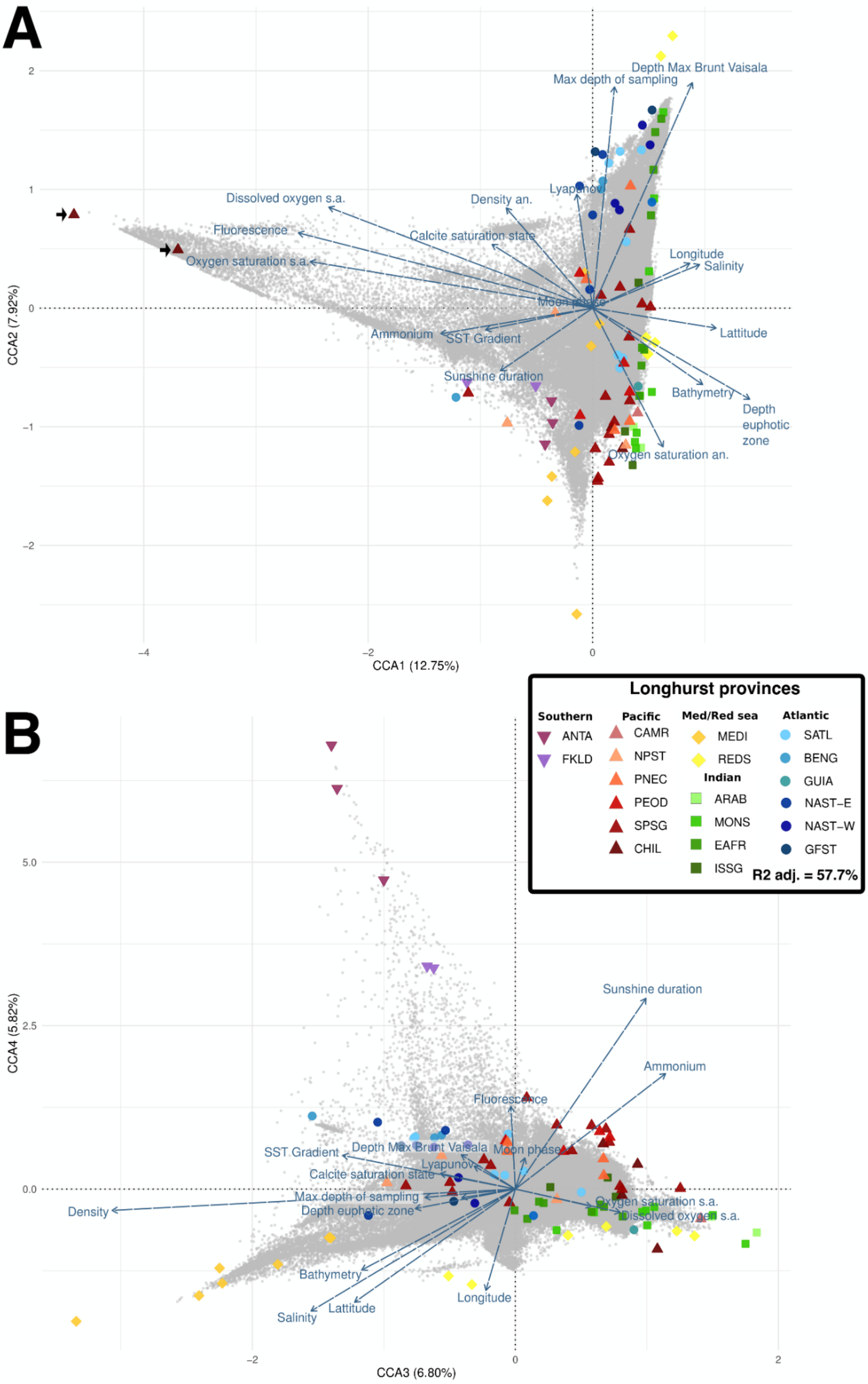

Supplementary Figure 5*: Canonical correspondence analysis (CCA) on abundances of the 130,650 protein functional clusters associated with models showing R^2 values over 0.25 . Panel A corresponds to the two first axes of the CCA, while panel B represents the third and fourth axes. In both panels, PFCs are represented as grey dots, quantitative environmental variables as blue arrows, and samples as points colored and shaped according to their biogeographical province (correspondence between 4 letters codes used here and full biogeographical provinces names, as well as descriptions of all other environmental variables are available in Supplementary Data 2). For simplification issues, other qualitative variables (Season moment, Size fraction and Ocean region) were not represented. The two samples from station 93 were highlighted by black arrows in panel A.*

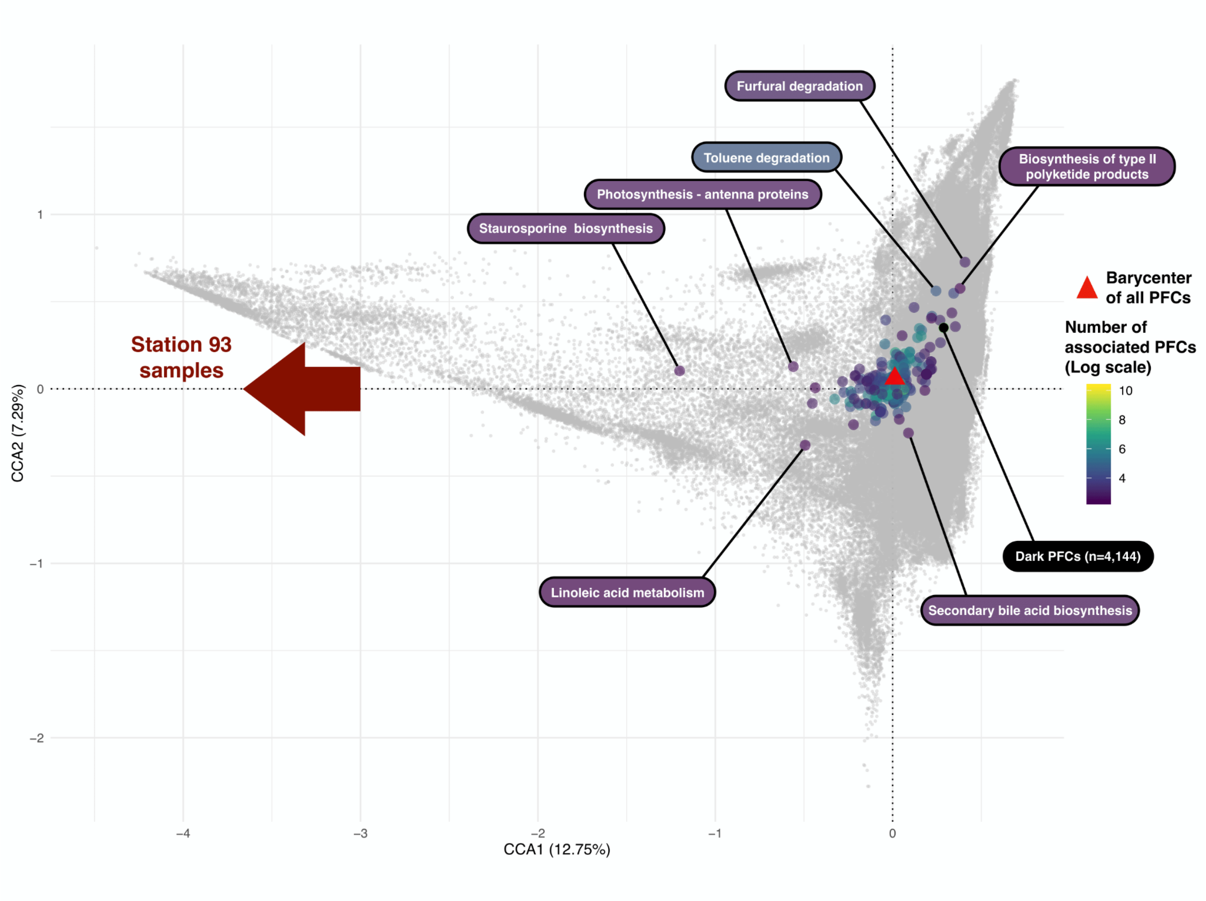

Supplementary Figure 6*: Barycenters of all metabolic pathways detected at least 10 times in the canonical correspondance analysis (CCA) space. Grey dots represent PFCs associated with models showing R^2 values above 0.25, in the same way as in Figure S5. Coloured dots represent the barycenters of each KEGG metabolic pathway detected at least 10 times, a pathway barycenter corresponding to the barycenter of the positions of all its associated PFCs. The colour of each barycenter codes for the number of its associated PFCs, in log scale. The barycenter of dark PFCs (no functional annotation and no taxonomic annotation under the Phylum level) was indicated in black. A colored arrow was represented to indicate the CCA side corresponding to the two outlier samples from station 93.*

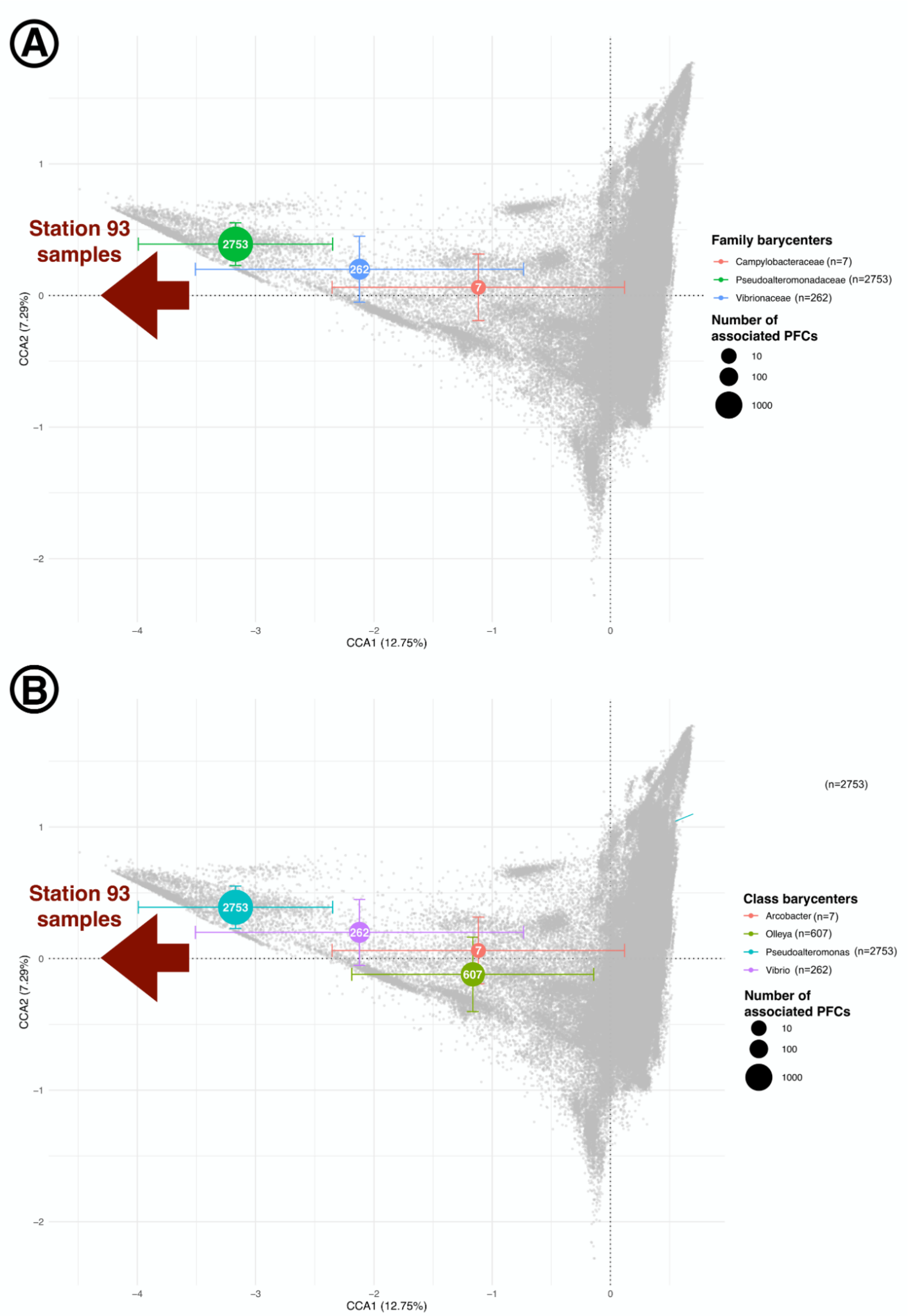

Supplementary Figure 7*: Distribution in the canonical correspondence analysis (CCA) space of the barycenters of protein functional clusters corresponding to models with R^2 values above 0.25 that were associated to particular taxa: (A) 3 selected family and (B) 4 selected genera. These taxa were selected because they were the only families and genera with barycenters below -1 on CCA1. Error bars correspond to the standard deviations of PFCs positions around their barycenters on CCA1 and CCA2 axes for each taxa. The size of barycenters represents the number of associated PFCs for each taxa, with the exact corresponding values written in white in each barycenter as well as in the legend. A colored arrow was represented to indicate the CCA side corresponding to the two outlier samples from station 93.*
