# Supplementary material for: Towards omics-based predictions of planktonic functional composition from environmental data
Source: Nat Commun. 2021 Jul 16;12:4361. doi: 10.1038/s41467-021-24547-1 (PMC8285379; doi:10.1038/s41467-021-24547-1)
Supplement: Supplementary file 3 — Description of Additional Supplementary Files [file 41467_2021_24547_MOESM3_ESM.pdf]

File Name: Supplementary Data 1

Description: List of Kegg metabolic pathways detected in protein functional clusters (PFCs). The number of occurrences in the 233,756 PFCs and in the 14,585 PFCs highly linked to environmental gradients (hlePFCs) are given for each pathway. The percentage of occurrences among hlePFCs is also given for each pathway, reflecting how the pathway was selected by our random forest approach.

File Name: Supplementary Data 2

Description: Description of the 52 environmental variables used in the study.
