# Supplementary material for: Towards omics-based predictions of planktonic functional composition from environmental data
Source: Nat Commun. 2021 Jul 16;12:4361. doi: 10.1038/s41467-021-24547-1 (PMC8285379; doi:10.1038/s41467-021-24547-1)
Supplement: Supplementary file 6 — Reporting Summary [file 41467_2021_24547_MOESM6_ESM.pdf]

nature research

Corresponding author(s): Emile Faure

Last updated by author(s): May 12, 2021

Reporting Summary

Nature Research wishes to improve the reproducibility of the work that we publish. This form provides structure for consistency and transparency in reporting. For further information on Nature Research policies, see our Editorial Policies and the Editorial Policy Checklist.

Statistics

For all statistical analyses, confirm that the following items are present in the figure legend, table legend, main text, or Methods section.

n/a	Confirmed	
☐	☒	The exact sample size (*n*) for each experimental group/condition, given as a discrete number and unit of measurement
☐	☒	A statement on whether measurements were taken from distinct samples or whether the same sample was measured repeatedly
☐	☒	The statistical test(s) used AND whether they are one- or two-sided<br>*Only common tests should be described solely by name; describe more complex techniques in the Methods section.*
☐	☒	A description of all covariates tested
☐	☒	A description of any assumptions or corrections, such as tests of normality and adjustment for multiple comparisons
☐	☒	A full description of the statistical parameters including central tendency (e.g. means) or other basic estimates (e.g. regression coefficient) AND variation (e.g. standard deviation) or associated estimates of uncertainty (e.g. confidence intervals)
☒	☐	For null hypothesis testing, the test statistic (e.g. *F*, *t*, *r*) with confidence intervals, effect sizes, degrees of freedom and *P* value noted<br>*Give P values as exact values whenever suitable.*
☒	☐	For Bayesian analysis, information on the choice of priors and Markov chain Monte Carlo settings
☒	☐	For hierarchical and complex designs, identification of the appropriate level for tests and full reporting of outcomes
☒	☐	Estimates of effect sizes (e.g. Cohen's *d*, Pearson's *r*), indicating how they were calculated

Our web collection on statistics for biologists contains articles on many of the points above.

Software and code

Policy information about availability of computer code

Data collection	All the genomic data used in this study were retrieved from public databases using the code provided at http://merenlab.org/data/tara-ocean-mags/
Data analysis	We used Prodigal v2.6.3, Salmon v0.11.3, Diamond v0.8.22, EggNOG mapper v4.5.1, KOFamScan v1.2.0, R v3.5.3., bash v4.3.48 and perl v5.22.1. All codes available at https://github.com/EmileFaure/MAGsProteinFunctionalClusters.

For manuscripts utilizing custom algorithms or software that are central to the research but not yet described in published literature, software must be made available to editors and reviewers. We strongly encourage code deposition in a community repository (e.g. GitHub). See the Nature Research guidelines for submitting code & software for further information.

Data

Policy information about availability of data

All manuscripts must include a data availability statement. This statement should provide the following information, where applicable:

- Accession codes, unique identifiers, or web links for publicly available datasets
- A list of figures that have associated raw data
- A description of any restrictions on data availability

Instructions on how to build or download the MAGs and metagenomes used in this study are available at http://merenlab.org/data/tara-oceans-mags/. Tools and databases used for functional annotations are available at http://eggnog-mapper.embl.de/ and https://www.genome.jp/tools/kofamkoala/. All other data used in this study are available at 10.6084/m9.figshare.12030795, including fasta files containing nucleotide sequences of all proteins in PFCs, hlePFCs, dark PFCs, PFCs associated with the Station 93, and PFCs associated with the Station 93 linked with Pseudoalteromonas MAGs. In this figshare repository, we also provide summary tables including all PFC and random forest associated statistics (e.g. all homogeneity and unknown scores, R2 values, variables importances) for each PFCs, hlePFCs and dark PFCs. Finally, we offer tables at the single protein level showing the PFC ID, taxonomic and functional annotations, and nucleotide sequences of each

protein in PFCs, hlePFCs and dark PFCs.

Field-specific reporting

Please select the one below that is the best fit for your research. If you are not sure, read the appropriate sections before making your selection.

☐ Life sciences ☐ Behavioural & social sciences ☒ Ecological, evolutionary & environmental sciences

For a reference copy of the document with all sections, see nature.com/documents/nr-reporting-summary-flat.pdf

Ecological, evolutionary & environmental sciences study design

All studies must disclose on these points even when the disclosure is negative.

Study description	We used random forest regression models to predict the abundance of 233,756 protein functional clusters from 51 environmental variables, in 93 samples from the global ocean. The 233,756 clusters were obtained through a sequence similarity network containing the 1,914,171 proteins of 885 prokaryotic metagenome-assembled genomes (MAGs). Our study is entirely based on published and publicly available data.
Research sample	Our study is based on 93 publicly available Tara Oceans metagenomes retrieved from 61 surface samples and 32 deep chlorophyll maximum samples collected worldwide in the global ocean, using a size filter targeting free-living prokaryotic microorganisms (0.2-3 µm). The original TARA Oceans metagenomes are available through the European Bioinformatics Institute (ERP001736) and NCBI (PRJEB1787).
Sampling strategy	The complete Tara Oceans sampling strategy was described in : Pesant, S., Not, F., Picheral, M., Kandels-Lewis, S., Le Bescot, N., Gorsky, G., ... & Dimier, C. (2015). Open science resources for the discovery and analysis of Tara Oceans data. Scientific data, 2(1), 1-16.
Data collection	No data collection was specifically designed for this study, as all data came from published Tara Oceans datasets. Seawater was filtered from different depths to retain small cell sizes (prokaryotic organisms). The DNA was extracted and submitted to high throughput sequencing. Data collection and recording on the EBI and NCBI was achieved by the Tara Oceans consortium, as described in Pesant, S., Not, F., Picheral, M., Kandels-Lewis, S., Le Bescot, N., Gorsky, G., ... & Dimier, C. (2015). Open science resources for the discovery and analysis of Tara Oceans data. Scientific data, 2(1), 1-16. and Alberti, A., Poulain, J., Engelen, S., ... Viral to metazoan marine plankton nucleotide sequences from the Tara Oceans expedition. Scientific Data, 4, 170093 (2017).
Timing and spatial scale	The circumglobal sampling took place during the Tara Oceans expedition, allowing to gather samples from more than 20 biogeographical provinces of the global ocean between 2009 and 2013. More details in Pesant, S., Not, F., Picheral, M., Kandels-Lewis, S., Le Bescot, N., Gorsky, G., ... & Dimier, C. (2015). Open science resources for the discovery and analysis of Tara Oceans data. Scientific data, 2(1), 1-16.
Data exclusions	No data were excluded from our analysis.
Reproducibility	Our analysis is entirely reproducible through the code and data made available on github and figshare.
Randomization	This is not relevant for our study, as we focused on environmental samples in which no groups or controls were defined.
Blinding	This is not relevant for our study, as we focused on environmental samples in which no groups or controls were defined.

Did the study involve field work? ☐ Yes ☒ No

Reporting for specific materials, systems and methods

We require information from authors about some types of materials, experimental systems and methods used in many studies. Here, indicate whether each material, system or method listed is relevant to your study. If you are not sure if a list item applies to your research, read the appropriate section before selecting a response.

Materials & experimental systems

n/a	Involved in the study	
☒	☐	Antibodies
☒	☐	Eukaryotic cell lines
☒	☐	Palaeontology and archaeology
☒	☐	Animals and other organisms
☒	☐	Human research participants
☒	☐	Clinical data
☒	☐	Dual use research of concern

Methods

n/a	Involved in the study	
☒	☐	ChIP-seq
☒	☐	Flow cytometry
☒	☐	MRI-based neuroimaging
